# The effects of reverse dieting on mitigating weight regain after a caloric deficit: a preliminary analysis

**DOI:** 10.1080/15502783.2025.2550185

**Published:** 2025-08-26

**Authors:** Valentina Rodriguez Da Silva, Mateus Muniz, Gretchen Shelton, Daniel Arevalo, Eric T. Trexler, Avinash Konapala, Landon Shannahan, Samuel Blanke, Grace Chong, Ryan Singh, Bill I. Campbell

**Affiliations:** a University of South Florida, Exercise Science & Kinesiology Program, Performance & Physique Enhancement Lab, Tampa, FL, USA; b Duke University, Department of Evolutionary Anthropology, Durham, NC, USA

**Keywords:** Energy availability, menstruation, nutrition, metabolic rate

## Abstract

**Background:**

Reverse dieting is a post-dieting strategy that involves the gradual increase of calories to slowly return to weight maintenance and mitigate weight regain. While a common strategy applied by coaches, minimal research has assessed how reverse dieting compares to other post-dieting protocols in mitigating weight regain.

**Methods:**

This was a randomized, parallel group study. Participants (*n* = 49) were resistance-trained males and females aged 18–50 years. Following a pre-diet phase to establish baseline weight and caloric intake, subjects completed a diet phase in which they lost 5% of their initial bodyweight. Upon reaching the weight loss target, participants began one of three post-diet strategies: a gradual weekly increase in calories of 8.5% for males and 11.7% for females (REV), immediate return to estimated maintenance calories based on the Hall equation (NIH), or ad libitum control (CON), all of which were followed for 15 weeks. Relative weight regain, defined as percent weight regained from the end of diet to the end of the post-diet phase, and weight gain efficiency, defined as the absolute weight regained (kg) divided by the increase in average daily caloric intake from the end of the diet to the post-diet phase, were compared across groups using analysis of variance (ANOVA) and post-hoc testing with Holm correction.

**Results:**

All groups regained weight during the post-diet phase. Mean relative weight regain was 3.68 ± 2.75% in REV, 2.73 ± 3.14% in NIH, and 1.30 ± 2.3% in CON. There were no significant differences in weight regain between the three groups (*p* = 0.053). Mean weight gain efficiency was 0.00364 ± 0.0028, 0.00311 ± 0.0040, and 0.00076 ± 0.00544, respectively. No significant differences in weight gain efficiency were observed among groups (*p* = 0.132).

**Conclusions:**

Post-dieting strategy did not significantly influence either relative weight regain or weight gain efficiency. Participants who followed a reverse dieting approach experienced greater relative weight regain compared to those who ate ad libitum, though this did not reach a level of statistical significance. While all groups experienced weight regain, none surpassed their initial 5% weight loss. These findings suggest that a gradual increase in calories through reverse dieting may not be more effective at minimizing weight regain than less structured approaches or immediately returning to newly estimated maintenance calories.

